# Engineered pediatric human muscle models the interferon-driven transcriptome of juvenile dermatomyositis

**DOI:** 10.1038/s41598-026-72125-6

**Published:** 2026-09-22

**Authors:** Devjanee Swain Lenz, Jeffrey A. Dvergsten, Jessica L. Turnier, Jessica Neely, Chioma Madubata, Christine Goudsmit, Sukanya Banerjee, Kaveh Ardalan, Sara Kandil, George A. Truskey, Lauren T. Covert

**Affiliations:** 1 Duke University School of Medicine, Durham, NC USA; 2 Duke Sequencing and Genomics Technologies Core Facility, Durham, NC USA; 3 University of Michigan Medical School, Ann Arbor, MI USA; 4 University of California, San Francisco School of Medicine, San Francisco, CA USA; 5 Duke University Pratt School of Engineering, Durham, NC USA

**Keywords:** Juvenile dermatomyositis, Type I interferon, Muscle, Tissue engineering, Transcriptomics, Pathway enrichment analysis, Diseases, Immunology

## Abstract

Juvenile dermatomyositis (JDM) is a rare pediatric autoimmune disease characterized by a strong type I interferon (IFN) signature in muscle. Mechanistic and therapeutic discovery has been limited by the lack of a clinically relevant disease model. To address this, we tested whether IFN treatment of a human muscle-derived microphysiological system (myobundles) recapitulates the transcriptional profile of JDM muscle. Principal component analysis of RNA sequencing from healthy and JDM muscle along with IFN-treated and untreated myobundles revealed separation of healthy and untreated samples from JDM and IFN-treated samples. JDM muscle exhibited 4,121 differentially expressed genes (DEGs, p_adj_<0.05) compared to healthy muscle, of which 56.2% showed concordant differential expression in IFNβ-treated and 30.0% in IFNα-treated myobundles. Hallmark pathway analysis demonstrated concordant directional changes between JDM muscle and IFN-treated myobundles. IFN and inflammatory pathways were most upregulated; oxidative phosphorylation, fatty acid metabolism, and adipogenesis were downregulated, though not reproducible across sites. Normalized enrichment score correlations between JDM muscle and IFN-treated myobundles were strongly positive (*r* = 0.63, *p*=1 × 10^− 6^ for IFNα; *r* = 0.67, *p* = 1.3 × 10^− 7^ for IFNβ). These findings indicate that IFN-treated myobundles, particularly with IFNβ, mirror key transcriptional features of JDM muscle and provide a needed muscle-centric platform to study IFN-driven mechanisms and therapeutic targets.

**Supplementary Information:**

The online version contains supplementary material available at https://doi.org/10.1038/s41598-026-72125-6.

## Introduction

Juvenile dermatomyositis (JDM) is the most common idiopathic inflammatory myopathy of children, typically presenting as proximal muscle weakness and rash^1^. It is an autoimmune disorder of childhood onset that can involve other organs as well, such as the lungs, central nervous system, gut, joints, and heart^2,3^. The annual incidence of JDM is approximately 3 per million in the United States^4^. Therapies are broadly immunosuppressive, nonspecific, and often not well tolerated. Mechanistic insight and therapeutic discovery thus far have been limited by the rarity of the disease, the infrequent need for muscle biopsy, and the absence of accurate disease models.

Although inflammatory T and B cell infiltration of muscle is a prominent pathological feature and up to 70% of JDM patients have myositis-specific antibodies (MSAs) implicated in disease pathophysiology^2,5,6^, accumulating evidence suggests that skeletal muscle itself actively contributes to local inflammation and disease persistence. Muscle tissue from JDM patients exhibits marked upregulation of type I and II interferon (IFN)-stimulated genes (ISGs)^7^, along with increased expression of proinflammatory cytokines and chemokines that promote immune cell recruitment and trafficking^8^. Further, myogenic progenitor cells have been identified as a source for type I IFN and proangiogenic mediators^9^, suggesting an autocrine signaling loop may impair muscle proliferation and contribute to sustained weakness^10^.

Despite the increasingly apparent role of skeletal muscle in JDM pathogenesis, methods to study JDM muscle in vitro have been limited. To date, JDM research has relied on 2D cell culture and animal models. Two-dimensional cell culture models fail to capture the complex cell-cell interactions of a human tissue, even when composed of human cells. Other disadvantages include an inability to display tissue functions like muscle contraction and dissimilarities in spatial organization between cells on rigid plastic vs. in a living tissue. In addition, animal models, particularly murine models of myositis, may not match human disease severity, phenotype, or genetic basis^11^. Furthermore, no existing animal model develops pediatric-onset myositis with the phenotypic and histopathologic features of JDM^12,13^.

To address these research challenges, we have used a human muscle-derived microphysiological system, termed myobundles, to define the direct effects of type I IFNα and IFNβ on pediatric human muscle. Myobundles recapitulate the striated, multi-nucleated myofiber architecture of human skeletal muscle and can contract spontaneously or in response to electrical stimulation simulating nerve conduction. Importantly, myobundle response to soluble factors, such as cytokines and drugs, mimics physiological responses^14–18^. In the context of JDM, we have previously shown that IFNβ-treated myobundles demonstrate reduced contractility, increased MHC class I and myositis-specific antigen expression, altered proliferation and differentiation, and a robust proinflammatory transcriptomic signature^19–21^. These features recapitulate key pathological characteristics of JDM muscle, supporting the relevance of this model for studying IFN-driven muscle pathology. Additionally, cytokine profiling of IFN-treated myobundle supernatants revealed overlap with the inflammatory signature observed in sera from JDM patients, further implicating muscle as an active contributor to both local and systemic inflammation^22^.

While prior in vitro studies of IFN effects on muscle, including our own, have characterized how muscle cells respond to type I IFN exposure^19–22^, whether an in vitro muscle model reproduces the transcriptomic profile of patient muscle has not previously been tested in JDM. Without this benchmark, it remains unclear which disease-associated processes such a model can validly be used to investigate. In this study, we directly compared the transcriptomic profiles of JDM muscle and healthy pediatric muscle to determine whether disease-associated transcriptional changes could be recapitulated by IFN treatment of healthy donor-derived myobundles. We hypothesized that IFN-treated myobundles would closely mirror the JDM muscle transcriptome, with residual differences reflecting contributions from non-muscle cell populations absent from the myobundle system. To test this, we performed RNA sequencing on JDM and healthy muscle biopsies and on IFN-treated and untreated pediatric myobundles, enabling a direct assessment of the extent to which this human muscle-derived model captures core molecular features of JDM muscle.

## Methods

### Sex as a biological variable

Our study included muscle samples from both male and female patients. Sex as a biological variable was not analyzed due to small patient sample size and because the sex of the pediatric donors used to generate the myobundles was unknown. Future studies with larger cohorts and defined donor sex will be needed to assess potential sex-specific differences.

### Human primary myoblast cell culture

Healthy pediatric human muscle tissue was obtained from three donors as surgical waste from orthopedic procedures for non-myopathic diagnoses under Duke IRB approved protocols. Myoblast isolation, expansion, and cryopreservation followed our previously published protocol^14,23^. In summary, minced muscle was rinsed in PBS and digested in 0.05% trypsin for 30 min. Tissue was pelleted by centrifugation, pre-plated for 2 h, and then seeded onto Matrigel-coated flasks (Corning, Glendale, AZ, USA). Growth medium consisted of low glucose DMEM (Thermo Fisher Scientific, Waltham, MA, USA), supplemented with EGF (VWR, Radnor, PA, USA), fetuin (Millipore, Burlington, MA, USA), dexamethasone (Sigma-Aldrich, St. Louis, MO, USA), gentamicin and amphotericin B (both Thermo Fisher Scientific), and 10% fetal calf serum (Hyclone, Logan, UT, USA). Cells from each donor were banked at passage 2 in growth medium with 10% DMSO, then thawed and used for myobundle fabrication at passage 4.

### Human myobundle fabrication, culture, and treatment

Myobundles were fabricated as previously described^14,19^. Each construct was seeded with 7.5 × 10^5^ myoblasts from a single donor, suspended in a fibrinogen, Matrigel, thrombin mixture prepared in growth medium (GM) containing 1.5 mg/mL 6-aminocaproic acid. This suspension was dispensed into custom polydimethylsiloxane molds, cast from Teflon masters and pre-treated with Pluronic, around the beams of a porous nylon frame (Cerex Advanced Fabrics, Cantonment, FL, USA). After 30 min of polymerization at 37 °C, myobundles received GM and were maintained on a rocker at 37 °C, with daily medium exchange for 4 days. Myobundles were then removed from the molds and transferred to differentiation medium, which consisted of low glucose DMEM, 1% N2 supplement (Thermo Fisher), gentamicin, amphotericin B, and 2 mg/mL 6-aminocaproic acid. They then differentiated over 7 days on a rocker at 37 °C, with differentiation media exchanged every other day. Mature myobundles subsequently received IFNα (Sigma-Aldrich, St. Louis, MO, USA) or IFNβ (PeproTech, Cranbury, NJ, USA) at 0 (control), 5, 10, or 20 ng/mL for 7 days, again with medium exchanged every other day.

### Muscle biopsy and clinical data collection

Participants were enrolled through the multidisciplinary juvenile myositis programs at Duke University (Site 1, 2018) and the University of Michigan Ann Arbor (Site 2, 2024). All specimens were collected from open surgical biopsy of the vastus lateralis performed for clinical indications. In the JDM group, biopsy was performed for diagnostic evaluation of suspected idiopathic inflammatory myopathy; control specimens were obtained from children biopsied for indications unrelated to inflammatory or neuromuscular myopathy, in whom JDM was excluded on histopathologic review. Only tissue surplus to diagnostic requirements was used for research, under IRB-approved protocols at each institution. Eligibility for the JDM group required age below 18 years, biopsy performed as part of clinical care, and a diagnosis established jointly by a pediatric rheumatologist based on characteristic clinical features^24–26^ and by a neuropathologist based on muscle histopathology. Demographic and clinical variables were collected from the timepoint of biopsy.

### Experimental design and replication

Myobundles were generated from three healthy pediatric donors (all under 21 years of age; donor sex was not available), each fabricated as a separate batch. Three donors were used based on prior work from our group demonstrating detectable inter-donor transcriptional and functional variation at this number^19,20^. Biological replicates were defined as independent donors; technical replicates were defined as individual myobundles fabricated from the same donor and subjected to the same treatment condition. Two technical replicates per donor were sequenced for each IFN condition (IFNα and IFNβ at 5, 10, and 20 ng/mL), except for one donor at IFNα 10 ng/mL, for which only a single high-quality RNA sample was isolated. Untreated myobundles were sequenced in a greater number of technical replicates (*n* = 4, 7, and 3 across the three donors for a total of 14). In total, 49 myobundle RNA samples were analyzed (35 IFN-treated, 14 untreated) across two sequencing runs. Muscle biopsies comprised 20 samples (10 JDM, 10 healthy), divided between sites (Site 1 at Duke: 5 healthy, 4 JDM; Site 2 at University of Michigan: 5 healthy, 6 JDM). No formal power analysis was performed, as JDM is rare and the cohort represents all available biopsies meeting inclusion criteria at the participating sites; analyses are therefore exploratory and hypothesis-generating.

### RNA sequencing

RNA sequencing was performed at different institutions and times for the myobundles and muscle biopsies. RNA was isolated from flash-frozen cultured myobundles that had not undergone any contractile stimulation as previously published^20^ and approximately 5 mg of each frozen muscle biopsy sample using RNeasy Mini Kit (Qiagen 74104, Hilden, Germany). Samples were stored at -80 °C until assayed. For myobundles, the Sequencing and Genomic Technologies Core Facility (SGT) at Duke University (Site 1) used the Kapa stranded mRNA Kit (Roche Code: KR0960) to build cDNA libraries, which were pooled to equimolar concentration and subsequently sequenced with an Illumina NovaSeq 6000 to produce 50-bp paired-end reads. An average of 47.61 million reads was mapped per sample. RNA from the muscle samples collected at Duke University was degraded, with the Agilent Fragment Analyzer measuring RINs 4.2–9.3. Thus, the SGT used the Universal Plus Total RNA-Seq library preparation kit with NuQuant (Tecan 9156-A01) to build cDNA libraries. The SGT flagged two samples that were overamplified. All libraries regardless of overamplification were pooled at equimolar concentrations and subsequently sequenced with a NovaSeq 6000 to produce 100-bp paired-end reads. RNA from the muscle samples collected at University of Michigan (UM, Site 2) was processed by The Advanced Genomics Core at the University of Michigan. NEBNext UltraExpress RNA and NEBNext Poly(A) mRNA Magnetic Isolation Module (NEB #E7490) were used to build cDNA libraries, which were subsequently sequenced with an Illumina NovaSeq X Plus to produce 151-bp paired-end reads.

### Statistics

Duke RNA-seq data processing followed previously described workflows^20^. UM RNA-seq data processing used BCL Convert Conversion Software v4.0 (Illumina) to generate de-multiplexed Fastq files. The reads were trimmed using Cutadapt v2.3^27^ and were evaluated with FastQC^28^ to determine quality of the data. Reads were mapped to the reference genome GRCh38 (ENSEMBL 109), using STAR v2.7.8a^29^ and assigned count estimates to genes with RSEM v1.3.3^30^. Alignment options followed ENCODE standards for RNA-seq^29^. Gene identifiers were harmonized across datasets, which used differing nomenclature (Ensembl gene IDs and gene symbols), by mapping symbols to Ensembl 106 gene IDs. Genes reported under legacy NCBI symbols and genes split across alternate-haplotype contigs in the transcriptome index were resolved explicitly prior to merging. Analyses were subsequently restricted to genes measured at both sites. Counts were merged from all datasets at the gene level, including genes with at least 10 reads in at least three samples.

We used principal component analysis (PCA) to visualize the datasets. We normalized data using variance stabilizing transformation with the vst command and used prcomp to perform PCA. To determine PC significance, we compared the observed variance to that expected under a null distribution using Horn’s parallel analysis (PCAtools::parallelPCA, 100 permutations, threshold = 0.01). Given biological differences between in vitro myobundles and muscle biopsies, we performed two separate analyses for patient muscle and myobundles.

Differential gene expression was analyzed using the DESeq2 package in R with the apeglm shrinkage method to obtain stable log_2_FC values^31^. Adjusted p-values were calculated using the Benjamini–Hochberg false discovery rate^32^. We performed gene set enrichment analysis (GSEA) with fgsea to obtain normalized enrichment scores (NES) for the 50 Hallmark pathways^33^. To assess whether disease-associated signal was reproducible independently of collection site, we additionally performed differential expression and GSEA within each site separately, without batch correction, and compared the overlap of significant genes and the concordance of pathway enrichment between sites. For each PC, we compared signed gene loadings between biotype classes using a two-sided Wilcoxon rank-sum test, with effect size expressed as the probability of superiority (A = U/[n₁n₂]) and p-values adjusted across the eight tests by Benjamini-Hochberg. To calculate power of Site 1 relative to Site 2 a priori, we used the mean count for genes (Site 1 = 386, Site 2 = 512) and median estimated dispersion calculated from DESeq2 (Site 1 = 0.2295, Site 2 = 0.1423) to estimate power for log2FC = 2 and α = 0.05 in RNApower^34^. To calculate observed power of Site 1 relative to Site 2, we used the median lfcSE (Site 1 = 0.405, Site 2 = 0.283) to calculate the coefficients of variation of both sites by inverting the variance expression (cv = sqrt((lfcSE × ln2)² / (1/n₁ + 1/n₂) − 1/depth)) derived in Hart et al. and wrapped in DESeq2^34^. After determining site had a large but not biological effect on variance, we modelled gene expression on uncorrected counts as an additive function of site and condition (expression ~ site + condition). We additionally used ComBat-seq to remove batch effects of site in which patient samples were processed and repeated the analyses as above. Because applying a batch correction before differential testing does not account for the uncertainty introduced by the correction, we compared the results of the two approaches. Batch-corrected counts were used for visualization and for all analyses comparing muscle with myobundles, where a common expression scale across tissues was required. No batch correction was applied to the myobundle data, as myobundle PCA showed no run-associated separation; differential expression and GSEA were performed as described above. Myobundle differential expression was modelled as expression ~ treatment. Donor was not included as a covariate, and technical replicates were treated as independent observations. Gene and pathway scores (NES) were then compared between JDM muscle and IFN-treated myobundles, at p_adj_<0.05. Pearson correlations were calculated using the NES values. Effect sizes are reported as log_2_FC for differential expression and NES for pathway analysis.

To understand the strength of IFN signaling across myobundles and muscle samples, all biopsy and myobundle raw counts were combined. Because sequencing run was largely confounded with tissue type in this model, no batch correction was applied. We modeled gene expression as a single variable (~ group) and performed the same comparisons as above in a combined cross-tissue model. Additionally, we calculated IFN score, which is the mean log_2_FC for the following IFN-activated genes: ISG15, IFI6, MX1, RSAD2, MX2, OAS1, IRF9, IFITM1, OAS3, and IFI35^7^. IFN scores were computed from group-level contrasts rather than per-sample values; the distributions shown in Fig. 2c-d therefore represent variation across the ten constituent genes within a contrast, not variation across biological replicates, and no significance testing was performed on them.

## Results

### Disease and IFN treatment drive transcriptional clustering across muscle and myobundles

We obtained 10 JDM and 10 healthy pediatric control muscle biopsies and performed RNA sequencing on them across two separate sites. JDM patients were on average 9.3 years old (standard error of mean, SEM 1.2); 60% were female and 50% non-Hispanic white. Only one JDM biopsy was from a patient with ongoing immunosuppressive therapy with prednisone and methotrexate, having been diagnosed 2 years prior; the remaining 9 of the 10 JDM muscle biopsies were from treatment-naïve patients. Childhood Myositis Assessment Scale (CMAS) scores averaged 37 out of 52 (SEM 7.1) and Physician Global Assessment (PGA) scores averaged 6.3 out of 10 (SEM 0.9), indicating moderately active disease. Regarding MSA status, three patients were NXP2 antibody positive; one was Mi-2 antibody positive. Full clinical and demographic data can be found in Supplementary Table S1. Given the heterogeneity and multiple tissues in a muscle biopsy compared to the homogeneity of myobundles, we characterized the transcriptomes of muscle and myobundles separately to increase our power to detect biologically relevant gene expression changes in each group.

Because our samples came from two sources, we first assessed dataset quality. Biopsies were collected at two sites six years apart, and library preparation differed between them: Site 1 used total RNA-sequencing with rRNA-depletion, whereas Site 2 used mRNA-sequencing with poly(A) enrichment. We performed PCA on each dataset (Supplementary Fig. S1a). While samples from Site 1 partially separated by condition along PC1, which explained 33.3% of variance, samples from Site 2 clearly separated by condition along PC1, which explained 43.8% of the variance. We next examined the variance of the two datasets using normalization in DESeq2 (Supplementary Fig. S1b). At both sites, dispersion estimates and normalized counts scattered tightly around the fitted curve, indicating high-quality data. Consistent with the expectation that total RNA-sequencing is noisier than mRNA-sequencing^35^, the median gene-wise dispersion was 1.61x higher at Site 1 than at Site 2 (0.230 vs. 0.142, respectively). With the smaller sample size at Site 1 (*n* = 9 vs. *n* = 11), the higher dispersion inflated the standard error on log2FC by 1.4x relative to Site 2. Consequently, we predicted 51% power at Site 1 and 79% power at Site 2 to detect a log2FC of 2 for a given gene (α = 0.05).

Because PCA separated healthy and JDM samples, we next asked whether JDM samples from the two sites shared transcriptional profiles, using DESeq2. We filtered gene sets independently for each site and analyzed 19,162 genes at Site 1 and 19,759 at Site 2. We first confirmed the difference in power between the two sites. We queried the standard errors of log2FC for the 16,265 genes that were shared between the two sites and passed multiple filtering steps to produce a corrected P-value. As expected, Site 1 had less observed power than Site 2, although both sites had more power than expected (69% and 94%, respectively). Accordingly, Site 1 had a total of 689 differentially expressed genes (DEGs) between JDM and healthy patients (p_adj_ < 0.05), and Site 2 had 5,506 DEGs between JDM and healthy patients (p_adj_ < 0.05). Of the DEGs, 385 genes overlapped between the two sites with a 97.9% concordance in direction of log2FC. To ensure degradation did not directly influence the power of Site 1, we modeled expression as an additive function of RNA integrity and condition (~ RIN + condition). Log2FC estimates were nearly identical between models (rho = 0.997 without RIN, with 100% concordance in direction).

Because gene-level effects were directionally concordant, but the two sites differed in statistical power, we used GSEA against the MSigDB Hallmark gene sets to test whether the same pathways were enriched in the same direction at both sites (Supplementary Fig. S2). Of the 50 Hallmark gene sets tested, 20 were significantly enriched at Site 1 and 23 at Site 2, with 14 shared between sites with concordant direction in enrichment. All shared pathways were positively enriched except for myogenesis, which was concordantly downregulated. However, gene sets related to oxidative phosphorylation, peroxisome, MYC targets V1, fatty acid metabolism, and adipogenesis were negatively enriched at Site 2, yet were non-significantly changed and near neutral at Site 1. Despite these discrepancies, overall gene-level and gene set comparison revealed concordance, so we proceeded to analyze the datasets together to compare to our IFN-stimulated myobundle model.

In the combined muscle dataset, PCA showed that sequencing site dominated the variance (PC1 = 42.5%), with disease status explaining less (PC2 = 15.9%) (Supplementary Fig. S3). To test whether differing library chemistry accounted for this, we compared PC1 gene loadings across biotype classes. Consistent with rRNA depletion versus poly(A) selection, PC1 was stratified by biotype rather than disease: lncRNAs, pseudogenes, other non-coding RNAs, and immunoglobulin/T-cell receptor genes were all shifted toward Site 1 (p_adj_ = 8.5 × 10⁻^14^, 1.1 × 10⁻^3^, 3.9 × 10^-4^, and 1.3 × 10^-12^, respectively; Supplementary Table S2). In contrast, no biotype class was shifted by site on PC2, which instead separated healthy from JDM samples. Immunoglobulin and T-cell receptor genes were strongly shifted toward JDM samples on PC2 (A = 0.277, p_adj_ = 3.3 × 10^-28^; Supplementary Table S2), consistent with lymphocytic infiltration of JDM muscle and supporting PC2 as a disease axis rather than a technical one.

Given the effect of site on variance, we modeled gene expression as an additive function of site and condition (expression ~ site + condition). Of the 20,974 genes analyzed for differential gene expression, 1,621 genes were differentially expressed between JDM and healthy samples (p_adj_ < 0.05). Of the DEGs, 1,012 were upregulated and 609 were downregulated in JDM samples compared to healthy samples. We next assessed transcriptional coregulation with GSEA. Inflammatory pathways remained significantly enriched for upregulation in JDM patients (NES > 2.0, p_adj_ < 0.05). Oxidative phosphorylation was also significantly negatively enriched in the combined JDM cohort (NES < -2.0, p_adj_ < 0.05), indicating that pooling recovered a signal that was undetectable at Site 1 alone.

**Fig. 1:**
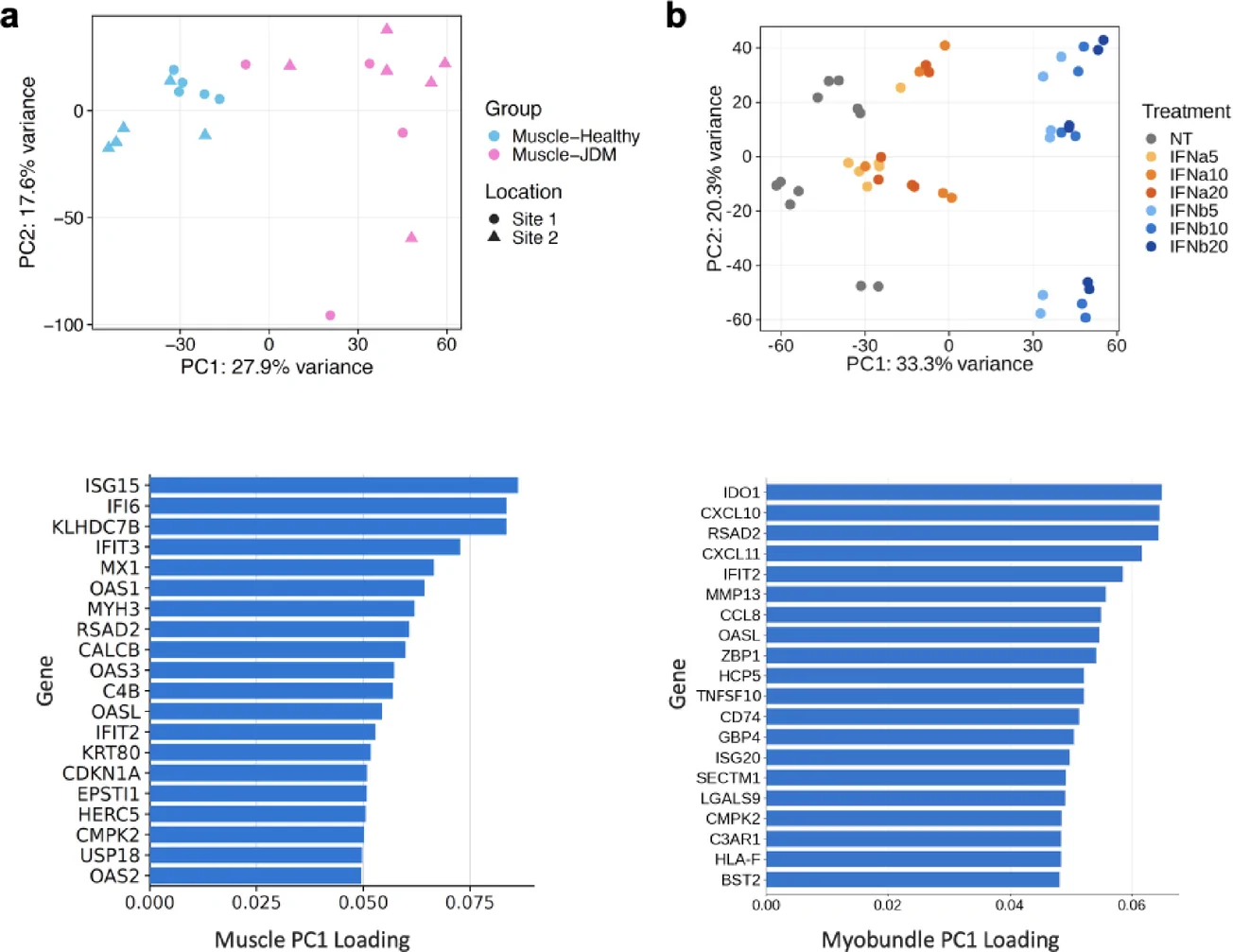
Principal component analysis of muscle biopsies and myobundles. (**a**) *Top*: PCA of batch-corrected transcriptomic profiles from healthy and JDM muscle biopsies shows separation along PC1 by disease status. *Bottom*: Genes with the highest loadings on PC1 primarily include interferon-stimulated genes (ISGs). (**b**) *Top*: PCA of myobundle transcriptomes separates samples along PC1 by interferon treatment, with IFNβ (in blue shades) inducing a greater rightward shift than IFNα (in orange shades) relative to untreated (NT) controls (grey). *Bottom*: Genes contributing most strongly to PC1 include ISGs, similar to those observed in JDM muscle biopsies. Muscle biopsies: *n* = 10 JDM and *n* = 10 healthy pediatric controls, batch-corrected using ComBat-seq. Myobundles *n* = 49 samples (derived from *N* = 3 healthy pediatric donors, *n* = 2 technical replicates per donor per IFN condition, except for one donor at IFNα 10 ng/mL with a single sample, and 14 untreated controls). PCA was performed on variance-stability counts, and PC significance was assessed by parallel analysis with 100 permutations.

We next analyzed the combined muscle dataset after correcting for site with ComBat-Seq. After correcting for site of sequencing, PCA showed that PC1 now separated samples by disease status, explaining 27.9% of the variance (Fig. 1a, top panel; scree plot associated with this PCA can be found in Supplementary Fig. S4a). Of the 20,974 genes analyzed for differential gene expression, 4121 genes were differentially expressed between JDM and healthy samples (p_adj_<0.05). Of the DEGs, 2,347 were upregulated and 1,774 were downregulated in JDM samples compared to healthy samples. To understand the impact of specific genes on principal components, we inspected the loadings of genes for PC1. The genes contributing the most to PC1 were primarily ISGs (Fig. 1a, bottom panel), consistent with the known upregulation of IFN signaling in JDM muscle.

We subsequently compared the transcriptomes of myobundles and muscle. Given the observed site-related differences are most likely due to differences in sequencing methods rather than underlying biological variation, we asked whether we could remove the effect of site on the dataset for our comparisons to our myobundle model. To ensure we were not overcorrecting the data and only removing technical artifacts, we compared our DESeq2 model of expression ~ site + condition to an analogous model of expression ~ condition using ComBat-Seq corrected data. Because differential testing on batch-corrected counts does not account for the uncertainty introduced by the correction, the corrected data yielded more significant genes than the covariate model (4,121 vs. 1,621 DEGs). The two approaches nonetheless agreed closely on the direction and rank of effects (rho = 0.92 for DEGs, rho = 0.99 for GSEA), and all directional and pathway-level conclusions below are supported by both. We therefore used the site-corrected dataset for analyses comparing myobundles and muscle, where a common expression scale across tissues was required.

We then characterized the transcriptome of healthy muscle-derived myobundles with and without IFN treatment. We analyzed the myobundles independently of muscle biopsies. PCA showed that 33.3% of the variance (PC1) was due to IFN treatment (Fig. 1b top panel; scree plot associated with this PCA can be found in Supplementary Fig. S4b). We examined the loadings of genes for PC1, and again, while the loadings of individual genes are small, genes with the greatest impact on PC1 were expectedly ISGs (Fig. 1b bottom panel). Additionally, consistent with our previous work^20^, IFNβ induced greater differential gene expression and stronger upregulation of IFN signaling compared to IFNα.

**Fig. 2:**
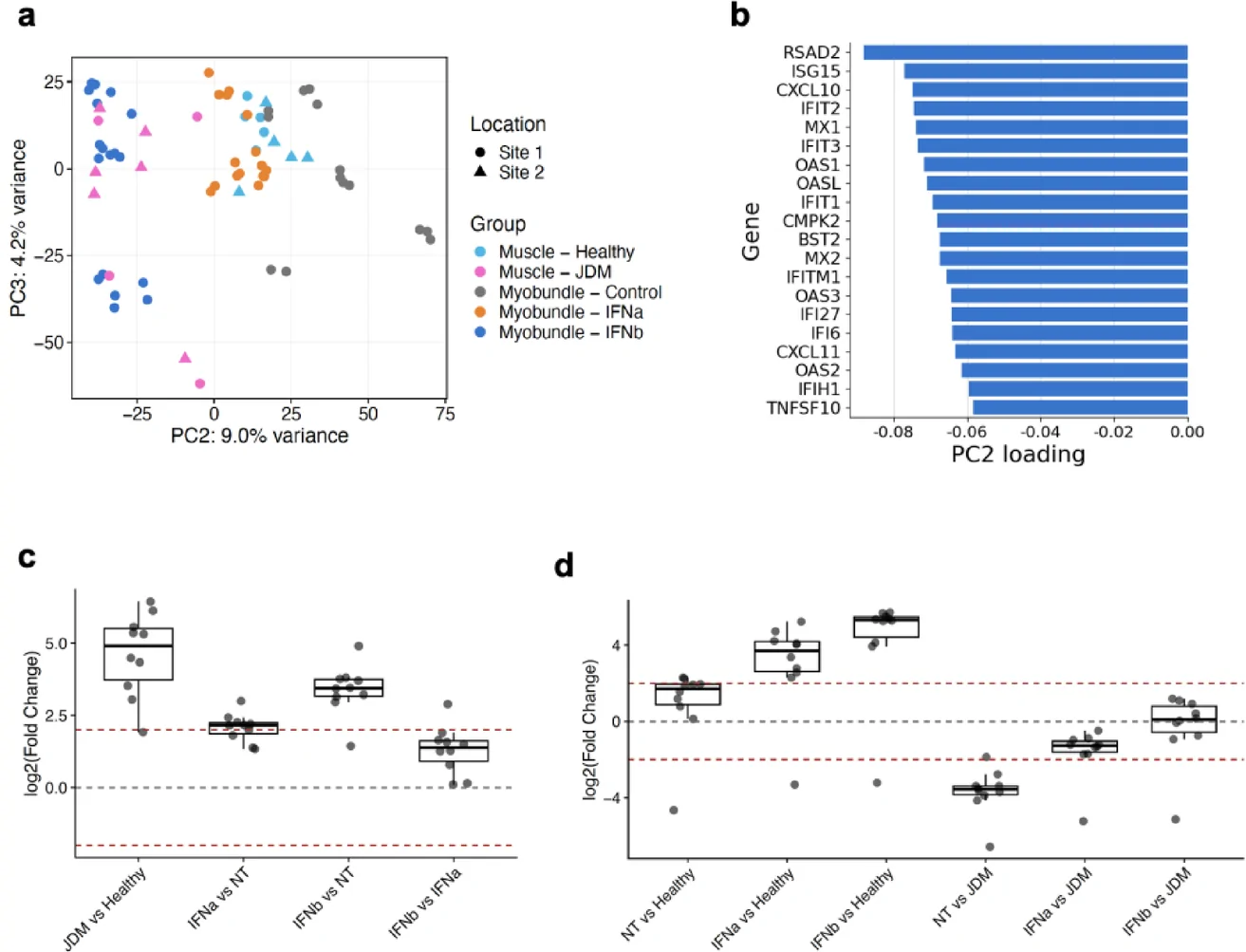
Comparison of JDM muscle and IFN-treated myobundles. (**a**) Principal component analysis of myobundles and muscle biopsies from two sites shows that PC2 separates JDM muscle from healthy muscle and IFN-treated myobundles from untreated myobundles. (**b**) Barplot of genes contributing most strongly to PC2 lists predominantly ISGs, all with negative loadings. This identifies PC2 as the type I IFN response axis on which more negative scores correspond to higher ISG expression. Both JDM muscle and IFNβ-treated myobundles shift toward negative PC2 values relative to their respective controls. (**c**) Log_2_FC of 10 interferon-stimulated genes. Each point is one of the ten genes; the central black bar is their mean (the IFN score). Distributions reflect variation across genes within a single group-level contrast, not across biological replicates, and are shown descriptively without significance testing. The grey dashed line represents no change, while the red dashed lines indicate |log_2_FC| *≥* 2. IFN scores are higher in JDM muscle and IFN-treated myobundles compared to controls. IFNβ treatment induces a greater ISG response than IFNα. (**d**) Comparison of IFN scores between muscle tissue and myobundles demonstrates comparable responses between healthy muscle and untreated myobundles, as well as between JDM muscle and IFN-treated myobundles. IFN-treated myobundles compared to healthy muscle expectedly show higher IFN scores. Muscle biopsies: *n* = 10 JDM and *n* = 10 healthy pediatric controls, batch-corrected using ComBat-seq. Myobundles *n* = 49 samples (derived from *N* = 3 healthy pediatric donors, *n* = 2 technical replicates per donor per IFN condition, except for one donor at IFNα 10 ng/mL with a single sample, and 14 untreated controls).

Given the strong effects of IFN signaling in both JDM muscle and IFN-treated myobundles, we performed a PCA of all samples (Supplementary Fig. S5a with associated scree plot in Supplementary Fig. S5b). PCA revealed distinct clustering by tissue type (muscle biopsy vs. myobundle) along PC1, which accounted for 65.1% of the total variance. Genes with the strongest loadings on PC1 towards muscle biopsies and away from myobundles were related to striated muscle contraction and mature muscle fibers (such as MB, MYL3, CASQ1) and endothelial markers not expected to be present in myobundles, reflecting differences in myofiber maturity and cellular composition between native muscle compared to myobundles (Supplementary Fig. S6). DEG counts (p_adj_<0.05) for all pairwise contrasts from the combined cross-tissue model, including comparisons between myobundles and native muscle, are provided in Supplementary Fig. S7. Because a common dispersion estimate is shared across all samples in this model, these counts are not directly comparable to the tissue-specific analyses shown in Fig. 4. Consistent with differences between tissue types, pathway differences between myobundles and native muscle are related to epithelial mesenchymal transition, protein secretion, and metabolic signaling, highlighting differences between cell culture and in vivo tissue. Notably, significance of IFNα pathway enrichment corroborates IFN score comparisons in Fig. 2d.

All sample principal component 2 (PC2) explained 9% of the variance and stratified samples according to disease and treatment status. JDM muscle biopsies exhibited a leftward shift relative to healthy controls; a similar shift was observed in IFN-treated myobundles compared with untreated controls (Fig. 2a). Notably, IFNβ-treated myobundles cluster and overlap more so with JDM muscle than IFNα-treated myobundles, which also separate from untreated controls. Top loading genes contributing to the shift along PC2 toward JDM disease status and IFNβ-treated myobundles were mostly canonical ISGs, including 8 of the 10 genes used to calculate IFN score (Fig. 2b).

To compare IFN signaling between myobundles and muscle, we calculated IFN scores for each group across the full dataset. As expected, JDM muscle exhibited higher IFN scores than healthy muscle, and IFN-treated myobundles had higher IFN scores than untreated myobundles, with IFNβ inducing higher scores than IFNα treatment (Fig. 2c). Compared to muscle biopsies, untreated myobundles exhibited IFN scores comparable to healthy muscle, and IFNβ-treated myobundles demonstrated similar IFN scores to JDM muscle (Fig. 2d). As expected, comparison of IFN-treated myobundles to healthy muscle showed higher IFN scores. Taken together, these findings indicate that transcription of IFN signaling in IFN-treated myobundles more closely resembles that of JDM muscle than that of healthy muscle.

### JDM muscle and IFN-treated myobundles have overlapping pathway enrichment

**Fig. 3:**
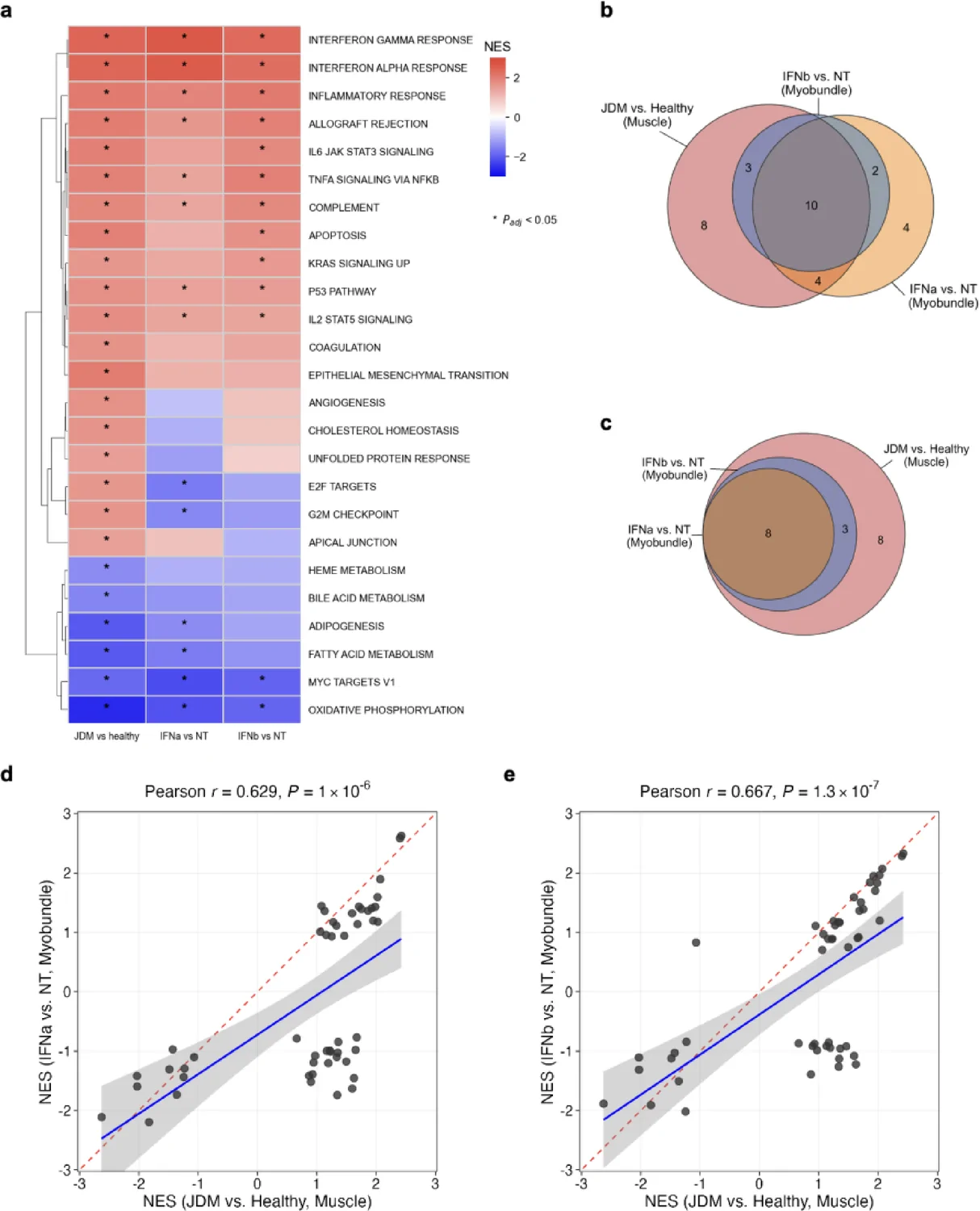
JDM muscle and IFN-treated myobundle concordance in pathway enrichment. (**a**) Heatmap of the 25 Hallmark pathways significantly enriched (padj < 0.05) in JDM vs. healthy muscle (*n* = 10 per group), with IFNα-treated vs. untreated (NT) myobundles, and IFNβ-treated vs. NT myobundles (*n* = 49 myobundles from three independent donors). Enrichment was assessed with fgsea across the 50 Hallmark gene sets with Benjamini-Hochberg correction. Rows were ordered by hierarchical clustering (Euclidean distance, average linkage). Color indicates NES; *asterisks denote padj < 0.05. IFNβ-treated myobundles showed more concordant pathway regulation with JDM muscle than IFNα-treated myobundles; only two pathways were discordant in direction (E2F targets, G2M checkpoint), reflecting enriched transcriptional programs in proliferating, regenerating cells in JDM muscle but not in myobundles. (**b**) Overlap of Hallmark pathways significantly enriched (FDR < 0.05) in the same three comparisons: JDM versus healthy muscle (red; 25 pathways), IFNα- versus NT myobundles (orange; 20 pathways), and IFNβ- versus NT myobundles (blue; 15 pathways). 13 of 15 IFNβ-enriched pathways are also enriched in JDM muscle. (**c**) Overlap of pathways significantly upregulated (FDR < 0.05, NES > 0) in the same comparisons showing all positively enriched pathways in IFN-treated myobundles overlap with JDM muscle. (**d**, **e**) Pathway-level concordance between JDM muscle and IFNα- (**d**) or IFNβ-treated (**e**) myobundles. Each point is one of the 50 Hallmark gene sets, plotted by NES in JDM versus healthy muscle (x-axis) against NES in IFN- versus NT myobundles (y-axis). Both correlations are positive, indicating that IFNα and IFNβ reproduce the direction of the JDM muscle pathway signature. Blue line, linear regression; grey band, 95% confidence interval; red dashed line, line of identity (y = x). NT = untreated.

Given IFN signaling genes contributed to PCs that explain variance due to disease or treatment state, we next examined whether IFN-related pathways were enriched for differential gene expression. As IFN signaling had a larger impact on PCA when tissues were split, we continued to analyze the datasets separately. We performed unbiased GSEA on the muscle and myobundle datasets individually to interrogate enrichment for differential gene expression in the 50 Hallmark pathways. Results demonstrated moderate-to-strong concordance in Hallmark pathway regulation between JDM muscle and IFN-treated myobundles (Fig. 3).

Among the 50 Hallmark pathways, 25 were significantly enriched in JDM vs. healthy muscle at p_adj_<0.05 (Fig. 3a). The most positively enriched pathways included IFNγ, IFNα, and inflammatory response pathways, which were all comparably and significantly upregulated in both JDM muscle and IFN-treated myobundles vs. controls. Other pathways with concordant positive enrichment included allograft rejection, IL6 JAK STAT3 signaling, TNFα signaling, complement, and apoptosis pathways. In contrast, E2F targets and G2M checkpoint pathways were significantly positively enriched in JDM muscle but negatively enriched in IFNα-treated myobundles, suggesting activation of proliferation-associated transcriptional programs from regenerating cells in JDM muscle tissue, but not in myobundles which lack regenerating satellite cells. Among the most significant negatively enriched pathways, oxidative phosphorylation, MYC targets V1, fatty acid metabolism, and adipogenesis were concordantly suppressed in both systems.

When comparing transcriptomic responses to IFNα and IFNβ in myobundles, many pathways were similarly regulated, including the expected upregulation of interferon-related and inflammatory pathways. However, several pathways showed discordant, non-significant regulation between the two type I IFNs; these included gene sets associated with angiogenesis, cholesterol homeostasis, unfolded protein response, and apical junction.

### IFNβ induces greater transcriptional change that more closely recapitulates JDM muscle

Concordance in pathway regulation was greater between JDM muscle and IFNβ-treated myobundles than between JDM muscle and IFNα-treated myobundles. Thirteen out of fifteen significantly altered Hallmark pathways in IFN$\:{\upbeta\:}$-treated myobundles overlapped with those altered in JDM muscle (Fig. 3b). While IFNα-treated myobundles had six pathways that were not significantly altered in JDM muscle, all significantly upregulated pathways overlapped with that of JDM muscle (Fig. 3c).

Consistent with this observation, GSEA revealed significant positive correlations between normalized enrichment scores in JDM muscle and IFN-treated myobundles. The correlation was stronger for IFNβ-treated myobundles (Pearson *r* = 0.67, *p* = 1.3 × 10^− 7^) than for IFNα-treated myobundles (Pearson *r* = 0.63, *p* = 1 × 10^− 6^) (Fig. 3d-e). As expected, for both IFNα- and IFNβ-treated myobundles compared to JDM muscle, pathways demonstrating strong concordance in upregulation included IFNγ and IFNα response and inflammatory response; strongly downregulated pathways that correlated included MYC targets V1, myogenesis and reactive oxygen species pathways. In contrast, a distinct subset of pathways did not correlate across myobundles and muscle; these included G2M checkpoint, E2F targets, mitotic spindle, angiogenesis, apical junction, and MTORC1 signaling pathways.

**Fig. 4:**
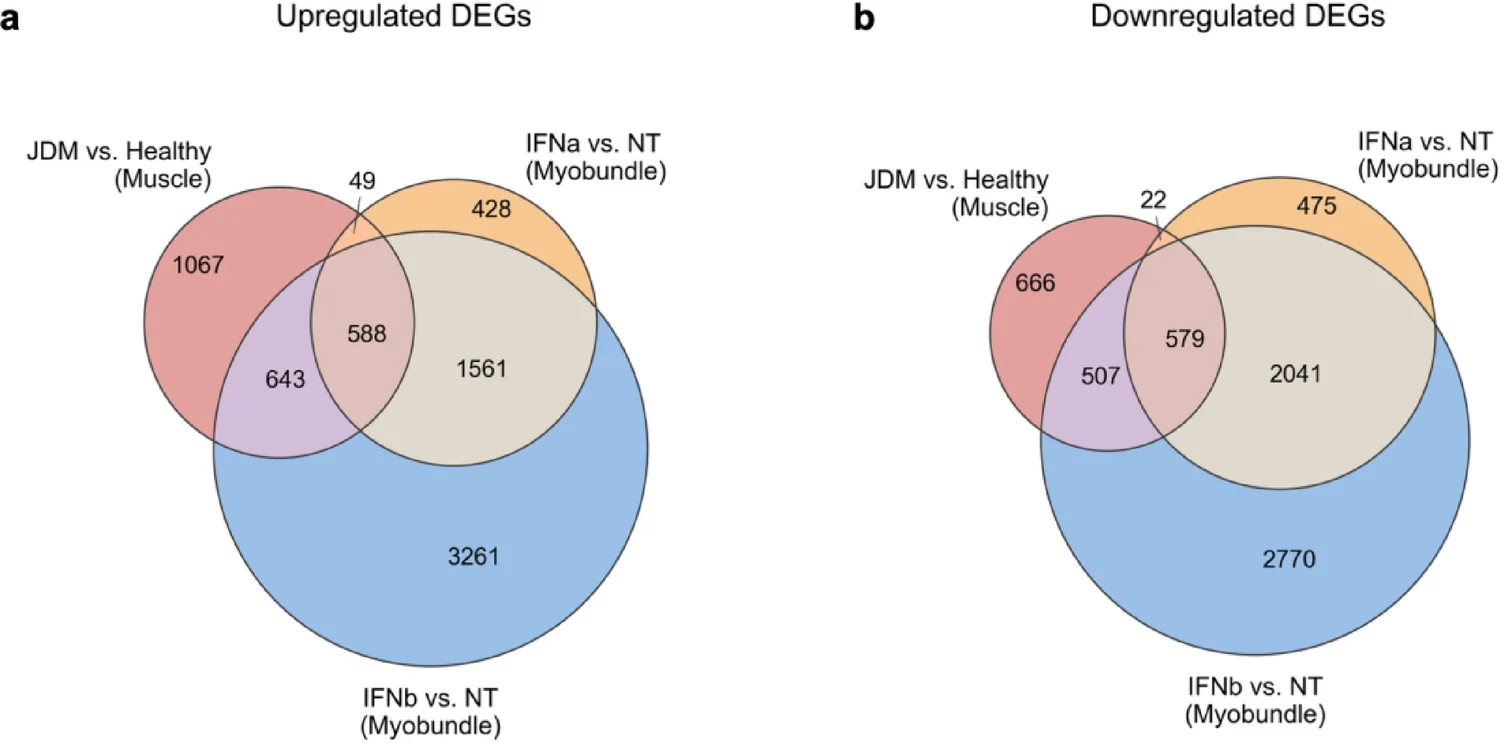
Overlap between differentially expressed genes in JDM muscle and IFN-treated myobundles. Differential expression was determined using DESeq2 with apeglm shrinkage, with significance defined as Benjamini-Hochberg p_adj_ < 0.05. DEGs were defined separately in each tissue: JDM versus healthy muscle biopsies, and IFN-treated versus untreated (NT) myobundles. IFNβ treatment yielded more DEGs and greater overlap with JDM muscle than IFNα for both upregulated (**a**) and downregulated (**b**) genes. Muscle comparison: *n* = 10 JDM vs. *n* = 10 healthy biopsies. Myobundle comparisons: IFNα- or IFNβ-treated vs. untreated myobundles from three independent donors.

IFNβ exposure also induced substantially greater transcriptomic remodeling in myobundles than IFNα treatment (Fig. 4). IFNβ-treated myobundles had 11,950 DEGs, approximately twice the number observed following IFNα treatment (5,743 DEGs). In comparison, JDM muscle displayed 4,121 DEGs relative to control tissue; notably, 56.2% of these were also differentially expressed in the same direction in IFNβ-treated myobundles, whereas only 30.0% overlapped with IFNα-treated myobundles. These findings suggest that IFNβ stimulation of myobundles elicits a gene expression profile that more closely mirrors that of JDM muscle than IFNα.

## Discussion

In this study, we explored how closely a human muscle-derived model reflected the molecular context of JDM. Prior IFN-based in vitro models, including 2D myotube systems and our own prior myobundle studies, characterized muscle responses to type I IFN but did not compare those responses against patient muscle transcriptomes; to our knowledge, this is the first direct comparison of an in vitro human tissue-engineered muscle model with ex vivo pediatric JDM muscle. We establish that IFN-treated pediatric myobundles reproduce the IFN-driven muscle-intrinsic components of the JDM transcriptome. IFNβ recapitulated JDM muscle more closely than IFNα, with concordant differential expression in 56.2% of JDM DEGs and stronger pathway-level correlation. Interferon, inflammatory, and cytokine signaling programs were reproducibly enriched in both patient muscle and treated myobundles, whereas vascular, regenerating, and immune cell-associated signatures present in patient muscle were absent from the model. The myobundle platform therefore provides a testable human tissue-based system for interrogating IFN-driven muscle pathology, while distinguishing disease programs that are muscle-intrinsic and IFN-inducible from those requiring additional cell populations the model does not contain.

No current in vitro or in vivo model of JDM, or idiopathic inflammatory myopathy (IIM) more broadly, fully represents the human disease^36^. Two-dimensional systems using primary or induced pluripotent stem cell-derived myoblasts and myotubes have advanced understanding of different myositis subtypes, particularly inclusion body myositis^37,38^, and murine models established by immunization, passive IgG transfer, or injury-induced inflammation capture features of anti-synthetase syndrome, immune-mediated necrotizing myopathy, and polymyositis^36,39,40^. None reproduces the childhood onset and muscle weakness that define JDM.

We have previously characterized this model across multiple domains. IFN-treated pediatric myobundles exhibit impaired contractile function, expression of myositis-specific antigens, and upregulation of MHC class I^19^. IFNβ exposure during muscle differentiation produces measurable injury and altered proliferation and differentiation capacity.^21^ Treatment with JAK inhibitors reverses IFN-induced transcriptional changes, demonstrating that the model is pharmacologically tractable^20^. IFN-stimulated myobundles also secrete a myokine profile resembling that observed in sera from treatment-naïve patients with JDM.^22^ The present study extends this validation to the transcriptomic level by directly comparing the model against JDM patient muscle. We note that transcriptomic concordance alone does not establish that the model reproduces JDM pathology; we recognize that it addresses one dimension of validation, complementing the functional, protein-level, and secretory characterization established previously. Integration of immune cell populations and mechanistic dissection of how IFN signaling produces contractile dysfunction are the focus of ongoing work.

An IFN gene signature is a well-established hallmark of JDM muscle and correlates with clinical disease severity^5,7,9,41–44^. Our comparative analysis of muscle biopsies from healthy and JDM patients confirmed a robust IFN gene signature in JDM muscle, with consistent transcriptomic shifts across all diseased biopsies collected at two independent sites. This IFN-associated transcriptional shift and increase in calculated IFN score were recapitulated in IFN-treated myobundles relative to untreated controls and healthy muscle. GSEA further demonstrated strong, reproducible upregulation of IFN-related and inflammatory pathways in both JDM muscle and IFN-treated myobundles. The overlap in transcriptomic change between ex vivo tissue and myobundles was characterized by concordant directionality and comparable magnitude of normalized enrichment scores and IFN scores.

In addition to expected concordant upregulation of IFN-associated pathways, we observed significant concordant downregulation of oxidative phosphorylation in both JDM muscle and IFN-treated myobundles. This is consistent with our prior work, in which expression of the most downregulated oxidative phosphorylation genes inversely correlated with IFN gene score; JAK inhibition partially reversed IFN-induced suppression of mitochondrial pathways^20^. Mitochondrial abnormalities in JDM muscle have long been described, from early ultrastructural observations^45^ through perifascicular COX-deficient fibers associated with MHC class I upregulation in up to 90% of children with JDM^46,47^, to transcriptomic studies linking IFN signaling and mitochondrial dysregulation^48^. In vitro work supports a direct effect, with type I IFN reducing mitochondrial respiration in human myotubes and acting synergistically with MHC class I overexpression to induce respiratory defects^49–51^. Our data are consistent with an IFN-associated contribution to these changes; however, as discussed below, metabolic pathway enrichment was not reproducible across our two sites, and we therefore regard this concordance as provisional.

IFNβ-treated myobundles exhibited greater transcriptional change and higher degree of overlap with JDM muscle than IFNα-treated myobundles. Although both IFNα and IFNβ signal through the same cell surface receptor complex (IFNAR1/IFNAR2) and induce largely overlapping ISG responses, their biological effects in muscle appear to differ. Notably, IFNβ, but not IFNα, induces contractile dysfunction in myobundles^19^, and IFNβ transcripts, but not IFNα transcripts, have previously been shown to be upregulated in JDM muscle by microarray analysis^42^. The molecular basis for this differential effect may lie in the substantially higher affinity of IFNβ for IFNAR1, estimated to be approximately 30-fold greater than that of IFNα^52^. This higher affinity is associated with prolonged STAT phosphorylation and sustained transcriptional activation of IFN-stimulated genes^53^, potentially explaining the greater transcriptomic shift observed with IFNβ exposure.

These findings have important therapeutic implications. While JAK inhibitors have demonstrated significant efficacy in JDM^54–56^, they broadly inhibit JAK-mediated signaling and thus block multiple cytokine pathways, including type I and II IFNs as well as various interleukins. In contrast, more targeted therapies, such as anifrolumab, a monoclonal antibody against IFNAR1, or the even more specific IFNβ-neutralizing antibody dazukibart, may offer improved efficacy with greater pathway selectivity. Dazukibart has already demonstrated proof-of-concept efficacy and safety in adults with moderate-to-severe dermatomyositis^57^ and 2 case reports of refractory JDM^58,59^. Our study supports the rationale for IFNβ-targeted therapies in JDM, as JDM muscle shows most overlap with IFNβ-induced transcriptomic changes in myobundles.

Although there is substantial transcriptional overlap between JDM muscle and IFN-treated myobundles, the absolute number of significantly differentially expressed genes is much higher in myobundles. This difference likely reflects gene expression changes associated with adaptive cell responses to the environment of in vitro culture and disrupted tissue organization^60^. While donors have transcriptional signatures influenced by tissue compartment, age, and gender^61^, many regulatory programs are lost in culture. Nevertheless, three-dimensional culture systems preserve physiological gene expression better than traditional monolayer cultures^62^. Although PCA showed that tissue type (myobundle vs. muscle) expectedly accounts for large differences in gene expression, broad transcriptional patterns, including individual gene and pathway differences, show meaningful overlap in changes seen between JDM muscle and IFN-treated myobundles relative to controls.

While the most significantly enriched Hallmark pathways in JDM muscle showed concordant change in IFN-treated myobundles, pathways that were discordantly regulated may reflect differences in the composition of myobundles, which lack muscle satellite cells. Only two pathways, E2F targets and G2M checkpoint pathways, were significantly upregulated in JDM muscle yet downregulated in IFNα-treated myobundles. Both pathways relate to cell cycle progression, which in JDM muscle is likely driven by myofiber regeneration, a process dependent on satellite cells. Thus, these differences in pathway regulation between JDM muscle and IFN-treated myobundles likely reflect how myobundles differ from native muscle in cellular composition and maturation state.

Our findings are consistent with the limited transcriptomic data available from JDM muscle. Roberson et al. reported type I IFN activation, upregulation of antigen presentation genes, and decreased expression of cellular respiration genes in treatment-naïve JDM muscle biopsies, mirroring both the IFN and metabolic signatures observed here^63^. Similarly, spatial transcriptomic profiling of treatment-naïve JDM muscle identified IFN pathway activation as well as mitochondrial abnormalities as independent features^64^. Direct cross-cohort validation is constrained by the rarity of this disease; purely JDM muscle RNA-seq cohorts are small, consisting of three to four patients, while the largest myositis muscle transcriptomic datasets combine adult and juvenile dermatomyositis^65^. Within this constraint, the concordance of our findings with independently collected cohorts supports the robustness of the IFN signature we report.

Strengths of this study include the use of primarily treatment-naïve JDM muscle biopsies, which enabled capture of transcriptional changes associated with moderate to severe disease activity in muscle. By using this human pediatric muscle-derived microphysiological system and directly comparing IFN-treated myobundles with JDM muscle, we support the model’s relevance in studying JDM-related muscle pathology. RNA sequencing also enabled unbiased profiling at both the individual gene and pathway levels, and concordance was demonstrated in DEG overlap, pathway enrichment directionality, and quantitative correlation of enrichment scores. Finally, the transcriptomic recapitulation of IFN-driven changes observed in IFNβ-treated myobundles supports further mechanistic studies and provides a rationale for preclinical testing of specific IFN-targeted therapies in JDM.

Several limitations should be acknowledged. Muscle biopsies were collected and processed at two sites at different times, with differences in tissue handling and library preparation, resulting in substantial batch effects that required correction with ComBat-seq^66^. The IFN and inflammatory signatures central to this study were significantly and concordantly enriched at each site independently, indicating that batch effects did not obscure them. Metabolic pathways, however, were not reproducible across sites; peroxisome, MYC targets V1, adipogenesis, fatty acid metabolism, and oxidative phosphorylation were significantly negatively enriched at Site 2 but near neutral and non-significant at Site 1. Total RNA-seq at Site 1 and poly(A) selection at Site 2 capture mitochondrial and non-polyadenylated transcripts differently, a recognized source of apparent differences in oxidative phosphorylation gene expression. We therefore cannot exclude a technical contribution to the metabolic downregulation observed in the combined analysis and interpret these findings as hypothesis-generating, pending confirmation in cohorts processed with harmonized protocols.

Additionally, the myobundle model lacks regenerating satellite cells, non-muscle resident and infiltrating cell populations, vascular structures, extracellular matrix remodeling, and the systemic inflammatory environment of JDM. Consistent with this, immunoglobulin and T-cell receptor transcripts were among the strongest contributors to the disease axis in patient muscle, representing a component of the JDM signature that a myobundle containing only muscle cells cannot reproduce; incorporating additional cell populations is therefore a priority for future work. Myobundle replicates also derive from only three donors, and technical replicates from the same donor were treated as independent observations in the differential expression model. This inflates the effective sample size and means absolute DEG counts are not directly comparable between myobundles (*n* = 49) and muscle biopsies (*n* = 20), which differ in both replicate structure and dispersion; our conclusions therefore rest on the direction and relative magnitude of transcriptional change rather than on absolute gene counts. Finally, the patient cohort was modest, some clinical metadata were incomplete, and not all MSA subsets were represented, limiting detailed clinical correlation and generalizability in this heterogeneous disease. We recognize that transcriptomic concordance alone does not establish mechanistic relevance, which will require functional validation. Despite these limitations, the model provides a valuable human-relevant platform for isolating IFN-driven effects in muscle.

In conclusion, JDM muscle from patients with moderate to severe disease activity shares meaningful transcriptomic overlap with IFN-treated myobundles, particularly IFNβ-treated ones. In addition to increased IFN signaling and inflammatory pathway activation, we observed downregulation of metabolic pathways, which warrant further study using standardized processing to distinguish biological from technical contributions. These findings support the model’s relevance for investigating IFN-driven, muscle-specific pathways in JDM pathogenesis. Future myobundle studies could clarify disease mechanisms, accelerate biomarker discovery, and assess novel therapies preclinically.

## Supplementary Information

Below is the link to the electronic supplementary material.

Supplementary Material 1

## Data Availability

The data discussed in this publication have been deposited in NCBI’s Gene Expression Omnibus63 and are accessible through GEO Series accession number GSE243339 and GSE328720. Scripts to analyze data can be found at https://github.com/devjanee/Covert_JDM.
