# Supplementary material for: Engineered pediatric human muscle models the interferon-driven transcriptome of juvenile dermatomyositis
Source: Sci Rep. 2026 Sep 22;16:32500. doi: 10.1038/s41598-026-72125-6 (PMC13672058; doi:10.1038/s41598-026-72125-6)
Supplement: Supplementary file 1 [file 41598_2026_72125_MOESM1_ESM.docx]

**Supplementary Material**

**Engineered pediatric human muscle models the interferon-driven transcriptome of juvenile dermatomyositis**

**Devjanee Swain Lenz^1,2^, Jeffrey A. Dvergsten^1^, Jessica L. Turnier^3^, Jessica Neely^4^, Chioma Madubata^4^, Christine Goudsmit^3^, Sukanya Banerjee^1^, Kaveh Ardalan^1^, Sara Kandil^5^, George A. Truskey^5^, Lauren T. Covert^1*^**

^1^Duke University School of Medicine, Durham, NC, USA

^2^Duke Sequencing and Genomics Technologies Core Facility, Durham, NC, USA

^3^University of Michigan Medical School, Ann Arbor, MI, USA

^4^University of California, San Francisco School of Medicine, San Francisco, CA, USA

^5^Duke University Pratt School of Engineering, Durham, NC, USA

*Corresponding author: [Lauren.covert@duke.edu](mailto:Lauren.covert@duke.edu)

| **JDM Patient Characteristic** | **JDM (n=10)** | **Missing data (n,%)** |
| --- | --- | --- |
| **Age, yr (±SEM)** | 9.3 (1.2) |  |
| **Female, n (%)** | 6 (60%) |  |
| **Race & ethnicity, n (%)** |  |  |
| Non-Hispanic White | 5 (50%) |  |
| Hispanic | 2 (20%) |  |
| Black | 1 (10%) |  |
| Other | 1 (10%) | Black/White |
| Unknown | 1 (10%) |  |
| **CMAS (±SEM)** | 37 (7.1) | 5 (50%) |
| **CDASI-A (±SEM)** | 5.6 (2.8) | 5 (50%) |
| **MSA positive, n (%)** | 4 (40%) | 3 (30%) |
| NXP-2 | 3 (30%) |  |
| Mi-2 | 1 (10%) |  |
| None | 3 (30%) |  |
| **MAA positive, n (%)** | 1 (10%) | 4 (40%) |
| Anti-SSA/Ro | 1 (10%) |  |
| **Physician's Global (±SEM)** | 6.3 (0.9) | 4 (40%) |
| PGA muscle | 5.5 (0.5) | 8 (80%) |
| PGA skin | 3.5 (3.5) | 8 (80%) |
| PGA extramuscular (no skin) | 2.5 (2.5) | 8 (80%) |
| **Patient/Parent Global** | 6.2 (1.5) | 5 (50%) |

**Supplementary Table S1. JDM patient demographics and clinical features.** Clinical and demographic data of the JDM patient cohort from which muscle biopsies were obtained. These biopsies were compared to 10 healthy control muscle biopsies.

CMAS, Childhood Myositis Assessment Scale; CDASI-A, Cutaneous Dermatomyositis Disease Area and Severity Index Activity Score; MSA, myositis-specific autoantibody; MAA, myositis-associated autoantibody; PGA, Physician Global Score

**Supplementary Figure S1. Quality control metrics of Site 1 and Site 2 data.** (a) Principal component analyses of Site 1 (left) and Site 2 (right). (b) Dispersion plots of Site 1 (left) and Site 2 (right). N=9 Site 1, n=11 Site 2, VST-normalized counts.

**Supplementary Figure S2.** Heatmap of normalized enrichment scores (NES) for all Hallmark gene sets in JDM versus healthy muscle for each site. fgsea across 50 Hallmark gene sets, BH-corrected, asterisks = p_adj_<0.05.

**Supplementary Figure S3. Collection site drives transcriptomic variation in muscle biopsies prior to batch correction.** Muscle samples (n = 10 JDM, n = 10 healthy pediatric controls) separated by collection site along PC1, which accounted for 42.5% of the variance, indicating a pronounced batch effect, which was subsequently corrected using ComBat-Seq.

| **PC** | **Biotype** | **n** | **A** | **r** | **Shifted toward** | **p** | **p (BH corrected)** |
| --- | --- | --- | --- | --- | --- | --- | --- |
| PC1 | lncRNA | 2555 | 0.453 | -0.094 | Site 1 | 2.1e-14 | 8.5e-14 |
|  | pseudogene | 642 | 0.461 | -0.077 | Site 1 | 8.5e-4 | 1.1e-3 |
|  | other ncRNA | 267 | 0.434 | -0.133 | Site 1 | 2.0e-4 | 3.9e-4 |
|  | IG/TR | 212 | 0.356 | -0.288 | Site 1 | 5.0e-13 | 1.3e-12 |
| PC2 | lncRNA | 2555 | 0.521 | +0.041 | Healthy | 8.2e-4 | 1.1e-3 |
|  | pseudogene | 642 | 0.518 | +0.035 | Healthy | 1.3e-1 | 1.3e-1 |
|  | other ncRNA | 267 | 0.557 | +0.115 | Healthy | 1.3e-3 | 1.4e-3 |
|  | IG/TR | 212 | 0.277 | -0.447 | JDM | 4.2e-29 | 3.3e-28 |

**Supplementary Table S2. Gene biotype stratification of principal component loadings in the combined muscle dataset prior to batch correction.** Signed gene loadings from PCA of all muscle biopsies before ComBat-seq correction (n = 10 JDM, n = 10 healthy; Supplementary Fig. S3) were compared between gene biotype classes for PC1 and PC2. For each biotype class the table reports the number of genes, the probability of superiority (A = U/[n₁n₂], where A = 0.5 indicates no difference between classes), the rank-biserial correlation (r), the direction of the shift, and raw and Benjamini–Hochberg-adjusted p-values across all eight tests. Each biotype class was compared against all remaining genes. On PC1, long non-coding RNAs and pseudogenes were shifted toward Site 1 (p_adj_ = 8.5x10⁻¹⁴ and p_adj_ = 1.1x10⁻3, respectively), consistent with retention of non-polyadenylated and unstable transcripts by rRNA-depletion total RNA-sequencing at Site 1 and their loss under poly(A) selection at Site 2. No biotype class was shifted by site on PC2. Immunoglobulin and T-cell receptor genes (IG/TR) were strongly shifted toward JDM samples (A = 0.277, p_adj_ = 3.3x10^-28^), consistent with lymphocytic infiltration of diseased muscle. Biotype annotations were taken from Ensembl 106. lncRNA, long non-coding RNA; PC, principal component; rRNA, ribosomal RNA.

**Supplementary Figure S4. Scree plots for principal component analyses of muscle and myobundle transcriptomes.** (a) Scree plot associated with PCA of batch-corrected transcriptomic profiles from healthy and JDM muscle biopsies (n = 10 per group) shown in main text Figure 1a. PCs 1-4 retain significance. (b) Scree plot associated with PCA of myobundle transcriptomes (n = 49 myobundles from three healthy pediatric donors) as shown in main text Figure 1b. PCs 1-6 retain significance. PC significance was assessed by parallel analysis with 100 permutations.

**Supplementary Figure S5**. **Tissue type is the dominant source of variation across muscle biopsies and myobundles.** (a) Principal component analysis of all muscle biopsies across 2 sites of collection (n = 10 JDM, n = 10 healthy) and myobundles (n = 49 from three independent donors), including untreated controls and those treated with IFNα or IFNβ, revealed separation along PC1 based on tissue type (muscle vs. myobundle) explaining 65.1% variance across samples. (b) Scree plot of the above PCA, showing significance of PCs 1-6.

**Supplementary Figure S6. PC1 loading genes reflect differences in myofiber maturation between muscle and myobundles.** Genes with the strongest loadings on PC1 from PCA of muscle biopsies (n = 10 JDM, n = 10 healthy) and myobundles (n= 49 from three independent donors) analyzed together across the shared gene set; PC1 explains 65.1% of the variance. Twenty-six of the top 30 genes load negatively, toward the muscle biopsies. The negative-loading genes are mainly contractile markers of mature myofibers (MB, MYL3, MYLK2, CASQ1), which are more abundant in native muscle than in engineered tissue. Other negative loading genes include VWF and CDH5 (endothelial markers) and CD93 (endothelial and myeloid cell marker), which are not expected to be present in myobundles. PC1 therefore separates samples by maturation state and cellular composition, independent of disease status or IFN treatment.

**c**

**Supplementary Figure S7. Differentially expressed gene counts from the pooled cross-tissue model.** Raw counts from all muscle biopsies (n = 10 JDM, n = 10 healthy) and all myobundles (n = 49 from three independent donors) were combined and modelled in DESeq2 as a single grouping variable (~ group), with apeglm shrinkage and Benjamini–Hochberg correction (p_adj_ < 0.05). All pairwise contrasts were extracted from this single fit. **(a)** Contrasts within a tissue type: JDM versus healthy muscle, IFNα- and IFNβ-treated versus untreated (NT) myobundles, and IFNβ- versus IFNα-treated myobundles. **(b)** Contrasts between myobundles and native muscle, comparing untreated, IFNα-treated, and IFNβ-treated myobundles against healthy and against JDM muscle. Bars show total significant DEGs, partitioned into upregulated (red) and downregulated (blue). Note the differing y-axis scales between panels. Because a common dispersion estimate is shared across all samples in this model, counts differ from the tissue-specific analyses in Fig. 4, in which muscle and myobundles were fit separately. (c) GSEA analyses of myobundle and muscle comparisons highlight heterogenetic tissue pathways between cell culture and tissue, including differences in epithelial mesenchymal transition, protein secretion, and metabolic signaling.
